# Role of histamine as a putative inhibitory transmitter in the honeybee antennal lobe

**DOI:** 10.1186/1742-9994-3-22

**Published:** 2006-12-29

**Authors:** Silke Sachse, Philipp Peele, Ana F Silbering, Martin Gühmann, C Giovanni Galizia

**Affiliations:** 1 Institut für Biologie – Neurobiologie, Freie Universität Berlin, Königin-Luise Str. 28-30, D-14195 Berlin, Germany; 2 Lehrstuhl für Neurobiologie, Universität Konstanz, D-78457 Konstanz, Germany; 3 Max Planck Institute for Chemical Ecology, Department of Evolutionary Neuroethology, Hans-Knoell-Str. 8, D-07745 Jena, Germany

## Abstract

**Background:**

Odors are represented by specific spatio-temporal activity patterns in the olfactory bulb of vertebrates and its insect analogue, the antennal lobe. In honeybees inhibitory circuits in the AL are involved in the processing of odors to shape afferent odor responses. GABA is known as an inhibitory transmitter in the antennal lobe, but not all interneurons are GABAergic. Therefore we sought to analyze the functional role of the inhibitory transmitter histamine for the processing of odors in the honeybee AL.

**Results:**

We optically recorded the representation of odors before, during and after histamine application at the input level (estimated from a compound signal), and at the output level (by selectively measuring the projection neurons). For both, histamine led to a strong and reversible reduction of odor-evoked responses.

**Conclusion:**

We propose that histamine, in addition to GABA, acts as an inhibitory transmitter in the honeybee AL and is therefore likely to play a role in odor processing.

## Background

The antennal lobe (AL) of insects is the functional analogue of the olfactory bulb (OB) in mammals, which is the first central neuropil where information from the olfactory sensory neurons (OSNs) is processed. Both the AL and OB, consist of different neuron types that modulate and optimize the afferent input in a complex network. It has been shown that intrinsic inhibitory circuits within the olfactory bulb and the AL shape temporal and spatial aspects of the odor-evoked patterns to improve odor detection and discrimination [1-4]. However, little is known about the synaptic interactions among the olfactory neurons involved in odor processing in insects.

In the honeybee, approx. 60,000 OSNs [5] convey olfactory information to two categories of AL neurons, namely approx. 4000 local interneurons (LNs) [6] and 700–800 projection neurons (PNs) [7,8]. LNs branch exclusively within the AL, whereas PNs relay the olfactory information to higher order brain centers. Synaptic contacts between the sensory neurons, LNs and PNs are mostly located in olfactory glomeruli [9]. Each of the approximately 160 glomeruli represents an identifiable morphological and functional subunit, arranged in a single layer around the honeybee AL [10,11]. Similar to the olfactory system of lobsters and moths [4,12], honeybees have anatomically distinct classes of olfactory LNs [13]. The majority of them, heterogeneous LNs (hetero LNs), have a high density of dendrite branches in one particular glomerulus and sparser branches distributed across other glomeruli. Homogeneous LNs (homo LNs) distribute their branches more homogeneously over the whole AL. It is conceivable that these different LN types are involved in functionally distinct inhibitory networks to shape the odor responses of olfactory PNs. Indeed, in vertebrates [14] and lobsters [15,16] dual inhibitory pathways at the first synaptic level have been well characterized. In lobsters the existence of both GABA- and histaminergic inhibitory pathways has been reported [17], whereas in vertebrates both pathways are mediated by the inhibitory transmitter GABA [18].

We have previously postulated the existence of at least two separate inhibitory networks in the honeybee AL, which both shape the odor-induced PN responses [2]. One is sensitive to application of picrotoxin, a GABA_A _channel blocker might also block some other chloride channels. Another is picrotoxin-insensitive and contrast-enhances overlapping glomerular response profiles. The PTX-insensitive effect could be mediated by metabotropic GABA receptors. GABA_B _antagonists have been proven effective in the *Drosophila *AL [1]. However, a different transmitter in addition to GABA should not be excluded. In contrast to LNs of moths and cockroaches, where most of the LNs are GABA-immunoreactive [19,20], only a fraction has been shown to be GABAergic in the honeybee [6,21]. There are approximately 35 histamine-immunoreactive LNs in the honeybee AL [22], suggesting histamine as a possible candidate similar to the lobster's olfactory system. The existence of histaminergic neurons in the AL is not ubiquitous to insects; some species totally lack histamine in the AL (e.g. *Drosophila*), some have few neurons that also branch in other brain areas (e.g. locusts), and some have a small number of histaminergic LN neurons (e.g. cockroaches) [23-25]. Histamine receptors in the *Drosophila *eye are insensitive to picrotoxin [26]. Therefore at least part of the inhibitory interactions that are still visible during picrotoxin application in honeybees could be mediated by histamine as a neurotransmitter. We therefore analyzed the effect of histamine application to the honeybee AL by optically recording two different processing levels. We measured the effect of histamine application on odor-evoked responses of a compound signal that mainly reflects the afferent input to the AL [27] and of AL output neurons (PNs). The results provide first evidence that histamine may act as an additional inhibitory transmitter in the honeybee AL, besides the already established role of GABA.

## Results

**Figure 1:**
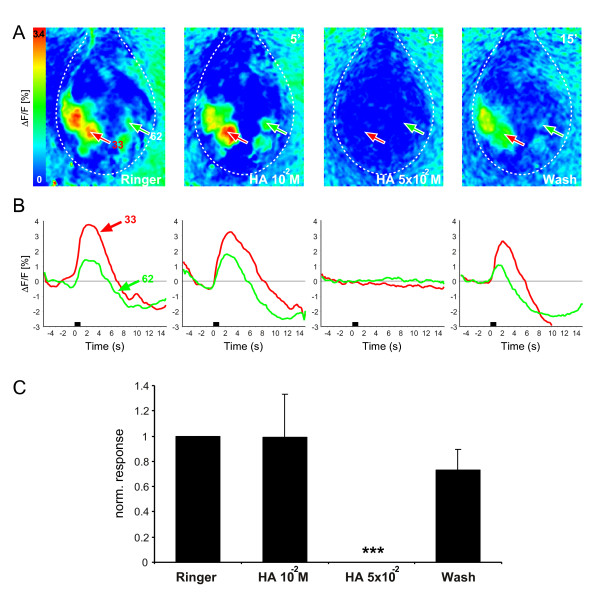
**Influence of histamine on the odor-induced compound responses of the honeybee AL (protocol 1)**. **A**: False-color coded spatial activity patterns to the odor 1-nonanol before, during and after histamine application. Histamine was successively applied with increasing concentrations. The AL border is marked with a dotted line, antennal nerve is at the top. The positions of two identified glomeruli are indicated in each frame. The numbers at the top right in each image indicate the time elapsed from the latest treatment change. **B**: Time traces of the two identified glomeruli, whose positions are marked in A. Odor application is shown by a black bar. A histamine concentration of 50 mM completely abolished the spatial and temporal calcium responses, which were reversible after wash-out. **C**: Bar chart of the odor-evoked responses averaged over all animals (mean and SEM, n = 7). Only the most-responsive glomeruli were included in the plot. The arrangement of the different bars from left to right reflects the temporal sequence of the experiment. Asterisks give significant differences to the Ringer measurement (***P < 0.001, two-tailed paired *t*-test, performed on the original data). The histamine effect observed for the animal in A and B was confirmed in each of the 7 animals tested.

We investigated the influence of histamine on the odor-evoked glomerular responses in the honeybee AL. We visualized different glomerular processing levels, using two different staining protocols in different animals. In protocol 1, we measured a compound signal after bath application with calcium green 2-AM, which emphasizes the afferent input to the AL (i.e. OSNs) [27,28]. In protocol 2, we selectively stained PNs using fura-dextran, thus measuring the AL output. Stimulation with odors led to strong, long-lasting and odor-specific calcium signals in several glomeruli in protocol 1 (Fig. 1A). The time courses of two identified glomeruli during stimulation with 1-nonanol are shown in Figure 1B. After application of 10 mM histamine to the brain, the calcium activity patterns remained unchanged, whereas 50 mM histamine totally abolished the odor-induced responses. In the wash the odor-specific calcium signals recovered, but appeared slightly reduced. The histamine effect observed in this animal was typical for all animals measured (n = 7; Fig. 1C). The reduction of the odor-evoked compound responses at 50 mM histamine was highly significant.

**Figure 2:**
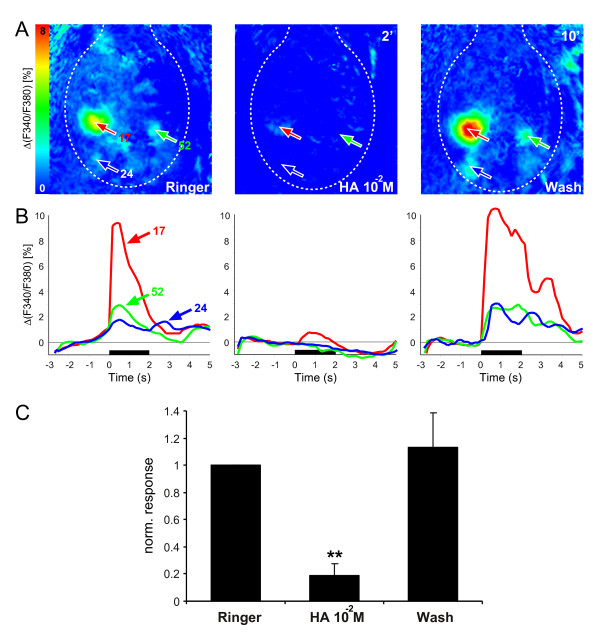
**Influence of histamine on the odor-induced PN responses of the honeybee AL (protocol 2)**. **A**: False-color coded spatial activity patterns to the odor 2-octanol before, during and after histamine application. Only one histamine concentration was tested in this animal. The AL border is marked with a dotted line, antennal nerve is at the top. The positions of three identified glomeruli are indicated in each frame. The numbers at the top right in each image indicate the time elapsed from the latest treatment. **B**: Time traces of the three identified glomeruli, whose positions are marked in A. Odor application is shown by a black bar. Contrary to the compound signals, PN responses were temporally complex and were strongly sensitive to a histamine concentration of 10 mM. The responses reappeared in the wash and were even stronger. **C**: Odor-induced PN responses averaged over all animals (mean and SEM, n = 5) of the most responsive glomeruli. Asterisks give significant differences to the Ringer measurement (**P < 0.01, two-tailed paired *t*-test, performed on the original data). Histamine significantly reduced the odor-evoked signals.

Similar to the compound signals (protocol 1), PNs (protocol 2) revealed a strong calcium increase following odor application (Fig. 2A,B). However, due to interglomerular processing these responses were temporally more complex compared to the compound signal [2,29,30]. PNs were spontaneously active and showed odor responses as published elsewhere [2]. For example, the odor 1-octanol elicited a weak on- and off-response (i.e. calcium increase after stimulus offset) in glomerulus 24; the latter is due to the release from inhibitory input. In contrast to the compound responses, application of histamine at a concentration of 10 mM strongly affected the PN signals: odor evoked calcium increases were abolished (Fig. 2A,B). In addition, spontaneous activity and odor-induced calcium-decreases were also abolished in all glomeruli (data not shown). In the wash a complete recovery of both spontaneous activity and odor responses could be observed. The histamine effect could be observed in all animals measured (n = 5; Fig. 2C). Concentrations lower than 10 mM did not influence the calcium signals in any animal measured (data not shown).

## Discussion

In this study we investigated the putative role of the transmitter histamine in the olfactory system of the honeybee. To this end, we optically recorded odor-evoked responses during histamine application either from the afferent input to the AL, estimated by a compound response (protocol 1), or from the AL output neurons (i.e. PNs, protocol 2). The results show that applying histamine to the honeybee brain led to a strong and reversible reduction of both the compound and the PN odor responses (Figs. 1, 2). Additionally, spontaneous activity of PNs was totally abolished due to histamine. These findings are in line with electrophysiological recordings of lobster olfactory neurons, which reported that histamine application suppressed both spontaneous and odor-evoked activity in OSNs [31,32] as well as electrically-induced responses in olfactory PNs [15]. Our applied histamine concentrations were 10- to 50-fold higher than described elsewhere [15]. However, those studies were carried out in isolated brain preparations in lobsters. In contrast we used an *in-vivo *preparation of the whole animal with an intact blood-brain barrier. It is likely that the blood-brain barrier has only a very limited permeability to histamine, which is a naturally occurring transmitter. Therefore, the effective histamine concentration at the cellular level is likely to be lower than the concentration applied to the whole brain.

Interestingly, higher concentrations of histamine were needed to silence the compound signal than the PNs. Several explanations could account for this difference. First, since the two signals were measured with different staining methods and different dyes, the observed shift could be due to a shift in recording sensitivity, rather than in the underlying physiology. Furthermore, since the two preparations differ, diffusion barriers may differ too. Second, it is conceivable that along the pathway from OSNs to PNs there are multiple occurrences of histaminergic channels. While the PN signal will be affected by all of them, the compound signal may be affected only by some. This may reduce the effect because of the resulting small numbers of synapses or also, because different populations of histaminergic receptors may have different affinities. The fact that histamine abolishes responses in the compound signal suggests that there is direct histaminergic input onto OSN synaptic terminals, such as is found in lobsters [33]. Cultured honeybee AL neurons (LNs and PNs) did not show any histamine-induced currents [34], further supporting the idea that OSN terminals express these receptors. However, that does not preclude the existence of histamine receptors on other cells than the OSNs, as stated above.

Histamine is the neurotransmitter in arthropod photoreceptors [35]. For the fly two histamine receptor types have been shown to be transmitter-gated chloride channels [26,36], similar to the histamine receptors found in lobster OSNs [37]. Moreover it is assumed that invertebrates lack metabotropic histamine receptors, and that histaminergic neurotransmission is exclusively mediated through ionotropic histamine receptors in invertebrates [38], further supporting the idea of an inhibitory effect by histamine-gated chloride channels. In the honeybee AL immunocytochemical studies showed approximately 35 histaminergic neurons [22]. Honeybees also possess the genes coding for histamine-gated chloride channels [39], but it is unknown whether these are expressed in the AL. Across-reaction of histamine with other receptors (e.g. GABA) is unlikely as shown in cell culture studies of honeybee AL neurons [34].

Taken together, our results provide physiological evidence that histamine may act as an inhibitory transmitter in the honeybee's olfactory system. Thus, we propose that GABA and histamine may be constituents of a multifaceted system of inhibitory transmitters in the AL, similar to findings in lobsters [15,16]. However, it is still unclear whether histaminergic and GABAergic LNs correspond to morphologically distinct LN types. Two types of LNs have been described in the honeybee: homo LNs and hetero LNs. Furthermore, different populations of LNs that express neuropeptides are known [40,41], adding to the complexity of the AL network. Further immunocytochemical experiments are needed to characterize GABA- and histaminergic neurons in the AL. Moreover, pharmacological experiments with histamine antagonists will help elucidating its role in odor processing in the honeybee's olfactory system.

## Methods

### Animal preparation and staining

Adult worker honeybees (*Apis mellifera*) were caught from different hives, quickly anesthetized by cooling and placed in a Plexiglas stage using dental wax. The antennae were fixed with silicone (Kwik-Sil™, WPI) at their scapus and covered with a coverslip. The head capsule was opened and glands and tracheae were carefully removed. In protocol 1, animals were then stained with calcium green to estimate OSN responses [27,28]. The brain was flooded with a solution of calcium green 2 AM (Molecular Probes, Eugene, OR; 50 μg dye was first dissolved in 50 μl Pluronic in DMSO and then diluted in 950 μl Ringer solution: 130 mM NaCl, 6 mM KCl, 4 mM MgCl_2_, 5 mM CaCl_2_, 160 mM sucrose, 25 mM glucose, 10 mM HEPES, pH 6.7, 500 mOsm; all chemicals from Sigma-Aldrich). After staining for 1 h, the brain was rinsed with fresh Ringer and the recording stage placed under the microscope. In protocol 2, PNs were selectively labeled with Fura to record PN responses as previously reported [2]. Briefly, a glass electrode, coated with Fura-dextran (potassium salt, 10,000 MW, Molecular probes), was inserted into the deutocerebrum aiming for the projection neurons (PNs) of the lateral antenno-cerebralis tract (l-ACT). The brain was then rinsed with Ringer solution to remove extracellular dye. After 3 h of staining, successful PN loading was visible by a strong staining of the l-ACT somata at the AL under the fluorescence microscope. Under these conditions, all signals measured in the AL came exclusively from PNs.

### Optical recording

Imaging was done using a T.I.L.L. Photonics imaging system (Martinsried, Germany). In case of calcium green, monochromatic excitation light was 475 nm, dichroic: 510 nm, emission: BP 515–565 nm, for Fura the excitation light alternated between 340 nm and 380 nm, dichroic: 410 nm, emission: LP 440 nm. Measurements were made with an upright microscope (Olympus BX 50WI), using 20× water immersion objectives (NA 0.95 for calcium green, NA 0.5 for Fura). Pixel image size was 2.4 × 2.4 μm. For each Fura recording a series of 60 frames was taken with a frequency of 6 Hz. Since odor-evoked compound signals measured with calcium green lasted longer than PN responses, we took recordings of 20 s (i.e. 100 frames with 5 Hz). Light was turned off between frames. Interstimulus interval was at least 1 min.

Odors were delivered to the antennae using a custom-made and computer-controlled olfactometer by switching from a constant air stream to an odor stream in order to eliminate mechanical stimulation [42]. Stimulus duration was 1 s for calcium green and 2 s for Fura measurements. Odors used differed between experiments, and were 1-hexanol, 2-octanol, 1-nonanol and linalool (Sigma-Aldrich). For each odor 4 μl of the odorant dissolved in mineral oil was applied to a filter paper (1 cm^2^) in a plastic syringe. Dilutions were adjusted to equalize effective vapor pressure for the different odorants. The control stimulus was a syringe plus filter paper with mineral oil.

Solutions of histamine (Sigma-Aldrich) were dissolved in Ringer for final concentrations of 10 mM and 50 mM and then bath-applied to the brain. In control experiments lower histamine concentrations (0.01, 0.1 and 1 mM) were also applied to the brain but no effects were observed (data not shown). Control experiments with histamine solutions controlled for pH (6.7) and osmolarity (500 mOsm; compensated by reducing the sucrose amount) gave identical results (n = 4, data not shown).

### Data processing

All analyses were done using custom software written in IDL (Research Systems, CO). The raw data were median-filtered for shot noise reduction (filter size 3 pixels in two spatial and one temporal dimension) and were corrected for scattered light by calculating an unsharp image with a radius of 50 μm and subtracting this from each frame. Calcium green signals were calculated as Δ*F/F *[%], where the mean of 19 frames measured before stimulus was used as *F*. These measurements were corrected for bleaching by fitting a logarithmic function computed for each measurement. In case of Fura-recordings, we calculated the ratio 340 nm/380 nm and multiplied it with 100; these values are labeled as Δ(*F*340/*F*380) [%] in the figures. Since each glomerulus had an individual background fluorescence ratio, all time traces were shifted to zero shortly before the stimulus onset by subtracting the background using frames 5–16 (i.e. before stimulus onset at frame 18). This allows a comparison between the traces of different glomeruli. We identified the strongest glomeruli on the basis of their response activities, using their published glomerular response profiles [2,43].

For the false-color coded images (Fig. 1A, 2A) we averaged the fluorescence changes between frames 25–45 for the compound responses (i.e. from stimulus onset until 3 s after stimulus offset) and frames 18–30 for the PNs (i.e. from stimulus onset until stimulus offset). For time courses of identified glomeruli (Figs. 1B, 2B) squares of 11 × 11 pixels (corresponding to 26.4 μm side length) were placed on the center of a glomerulus, their values were averaged and plotted against time.

We averaged the responses of the most activated glomeruli during Ringer, histamine and in the wash over all animals measured (n = 7 for calcium green, n = 5 for Fura; Figs. 1C, 2C). Beforehand, for each animal the glomerular response was calculated as the maximum during stimulus onset until 3 s after stimulus offset and repeated stimulations were averaged. In order to compare animals with different background fluorescence and thus different maximal activities, we normalized by defining the glomerular response within each animal to each odor before the pharmacological treatment as 1 and scaled the other responses accordingly. Significant differences were determined using a two-tailed paired *t*-test, performed on the original data.

## Competing interests

The author(s) declare that they have no competing interests.

## Authors' contributions

SS, PP, AFS, MG did the physiological measurements, SS did the data analysis, SS and CGG designed the study and wrote the manuscript.
